# Development of a laboratory-based frailty index for risk prediction in elderly trauma patients with hip fractures

**DOI:** 10.1007/s11357-025-01789-1

**Published:** 2025-07-17

**Authors:** Malou-Sophie Dietrich, Emmanouil Liodakis, Stephan Sehmisch, Marcel Winkelmann, Manfred Gogol

**Affiliations:** 1 Department of Trauma Surgery, Hannover Medical School, Carl-Neuberg-Straße 1, Hannover, 30625 Germany; 2 Department of Trauma Surgery, Hannover Medical School, Hannover, Lower Saxony Germany; 3 Department of Trauma, Hand and Reconstructive Surgery, Saarland University Medical Center, Homburg, Saarland Germany

**Keywords:** Hip fracture, Laboratory-based frailty index, Predictive tool

## Abstract

This study aimed to develop a laboratory-based frailty index (FI-Lab) to predict 12-month mortality risk in elderly patients following a hip fracture (HF). A retrospective analysis of 235 consecutive patients over 70 years old, who underwent HF surgery, was conducted. The FI-Lab, based on 21 routine blood parameters, was evaluated using Receiver Operating Characteristics, Area Under the Curve (AUC), Kaplan–Meier curves, and Cox proportional Hazard ratios. The FI-Lab showed an AUC of 0.7177 for 6-month and 0.7423 for 1-year survival. High FI-Lab values correlated with higher preoperative ASA scores, longer time-to-surgery times, more perioperative transfusions, and higher postoperative complication rates. The Cox hazard ratio revealed a significant increase in the risk of death for 1 year and 6 months from a score of 0.4 and higher. These findings highlight the FI-Lab’s clinical relevance and validity as a predictive tool, emphasizing the need for differentiated perioperative risk stratification.

## Introduction

The demographic shift, which is particularly evident in Western countries, means a rapid increase in the proportion of the elderly population. In Germany, the proportion of the population aged over 65 is expected to rise to 33% by 2060. Health outcomes in this age group vary due to different lifestyles and socioeconomic conditions, highlighting the urgent need to address age-related health challenges, particularly frailty, in older adults [1, 2]. Understanding which patients are at higher risk allows for better allocation of limited medical resources (e.g. ICU beds, nursing staff).

Frailty is a complex geriatric syndrome characterized by reduced resilience and increased vulnerability to stressors, and poses significant risks for older patients, particularly during hospitalization [3, 4]. The term “frailty” still lacks a precise definition, but categorizing frailty into different levels of severity allows for a more accurate classification of the health status of older patients so that they can be offered further diagnostics and targeted treatment [3, 5, 6]. Frailty is associated with diseases and functional decline as well as adverse outcomes such as falls, complications following medical procedures, loss of autonomy and increased mortality [7, 8]. As a dynamic process, it can impact treatment outcomes but is generally considered to be treatable and partially reversible [9]. Managing frailty in hospitals is a global challenge that requires tailored screening and therapeutic approaches [3, 10].

Osteoporosis associated fractures are prevalent reasons for admission to orthopedic departments among older adults and can lead to increased mortality, disability and high costs for the healthcare system with surgery being the main treatment option [11–15]. Patients’ responses to the fracture and treatment as major stressors may vary, affecting complication rates and rehabilitation success [16–18]. Further research is needed to improve the identification and management of frailty and to enhance outcomes for elderly patients with osteoporosis associated fractures. A major group of age-related fractures are hip fractures (HF). Patients with HF are difficult to screen due to immobility and high prevalence of cognitive decline. Limited time in the emergency department prior to surgery hinders comprehensive assessment. Existing frailty classification models, including the Physical Frailty Phenotype and the Accumulation of Deficiencies approach, provide valuable insights but are limited in their applicability, particularly in emergency settings. Fried et al.’s Physical Frailty Phenotype categorizes patients based on walking speed, hand grip strength, weight loss, activity, and fatigue [19]. Mitnitski and Rockwood’s Accumulation of Deficiencies model assesses over 70 criteria, measuring frailty on a scale [20].

The Nottingham Hip Fracture Score, developed for patients with hip fractures, considers factors such as age, gender, comorbidities, mental test results, hemoglobin levels, living situation, and malignancy to predict mortality risk within 30 days of a femur fracture. All these models are impractical in a fast-paced environment, especially for cognitively impaired patients [21].

Emerging approaches, such as laboratory-profile-based scoring systems, as developed by Ritt et al. using 22 blood and one urine parameter, show promise in providing rapid and practical frailty assessment tools, but have only been developed in post-acute general geriatric and ambulatory patients [3, 5, 22–25]. However, there is currently no practical tool for identifying high-risk trauma surgery patients with frailty in emergency settings. Developing alternative methods based on routine lab findings could be a promising approach for identifying frailty in this patient population.

This study aims to address this gap by developing a simple and rapid scoring system based solely on routinely collected laboratory parameters to predict mortality risk in orthopedic trauma patients without the need for active patient participation. Laboratory data from patients with HF were analyzed to develop and validate this scoring system, aiming to enhance its practicality and speed in clinical application.

## Methods

### Study design and study population

This retrospective cohort study was conducted at a Level 1 Trauma Center. All patients of both sexes aged 70 and over with HF who were surgically treated at the department of trauma surgery in a consecutive 13-month period were included. Patients were identified with their ICD10 diagnosis (S.72.0, S72.1, S72.2) within the hospital's data base. Clinical data were assessed from the electronic health records in the hospital information system (HIS). Patients with re-fracture during the observation period were not included again. Further exclusion criteria were the presence of < 70% of laboratory parameters, lack of access to data, refusal of a telephone interview and the inability to give written consent to participate in the study. The primary outcome was 1-year all-cause mortality. The 6-month survival was also considered as a secondary outcome.

Follow-up data were collected for the 1-year period following the patient’s operation. This was achieved by contacting the patients, authorized relatives, employees of the respective nursing home or general practitioners, and conducting a semi-structured telephone interview with them, in which 1-year survival and a comprehensive geriatric assessment were conducted using a standardized questionnaire. This assessment was carried out with the help of established measurement instruments, including self-reported health, the Whooley two-question test [26], the Parker Mobility Score (PMS) [27, 28], the Numerical Rating Scale (NRS), the European Quality of Life 5 Dimensions 5 Level (EQ-5D-5L) [29], level of care (German care group), number of rehospitalizations, and recurrent falls.

For secondary analysis, the data pertaining to the trauma surgery process (diagnosis, length of stay (LOS), discharge by location, surgical complications, use of anticoagulation, time in the emergency room, time to surgery (TTS), duration of surgery, type of surgery, perioperative transfusions) and general data (height, weight, Body Mass Index (BMI), American Society of Anesthesiologists (ASA), PMS on admission, level of care on admission) were included.

The study followed the principles of the declaration of Helsinki and good clinical practice. The study protocol was approved by the local ethical committee (No.11522_BO_K_2024).

### Creation of the FI-Lab

Routinely assessed laboratory data on admission with standard values for women and men are displayed in Table 1. The laboratory analysis was performed in the hematology and clinical chemistry laboratories at Hannover Medical School (HMS), providing a 24/7 service. All parameters were extracted from the HIS. Only parameters that were determined on arrival at the emergency room were included in the analysis. All laboratory parameters were coded as 0 or 1. A value of 0 indicates that the parameter is within the normal range and does not represent an abnormality. Conversely, a value of 1 indicates that the parameter is outside the normal range, either above or below it. The score is calculated by dividing the number of deviations present in each patient by the total number of possible deficits. This results in a value between 0 and 1 for each patient, with a value of 0 indicating that all parameters are within the normal range and a value of 1 indicating that all parameters deviate from the norm. In the case of missing parameters, the number by which they were divided was reduced by one point. Except for the estimated glomerular filtration rate (eGFR), the gender-specific reference parameters of the hospital were used for coding. For eGFR, the reference values were adjusted according to age. In place of a cut-off value of 90 ml/min, a cut-off value of 45 ml/min was employed in accordance with stage 3a of the staging of chronic kidney disease [30–32].

### FI-Lab44

The first step in the creation of the FI-Lab44 was to demonstrate a correlation between laboratory parameters and patient survival. To this end, all parameters of the Geriatric Trauma Center routinely collected on admission of the patient were utilized. This encompasses 44 parameters of various functional systems and organs, as detailed in Table 1.

### FI-Lab21

To create the FI-Lab21, the 13 parameters that were significant in the Volcano Plot were initially selected, and then a further 8 parameters with a (descending) tendency towards significance were included, which cover various organ systems: Hemoglobin, hematocrit, erythrocytes, red blood cell distribution width, mean corpuscular volume (MCV), 1.25 vitamin D, calcium, C-reactive protein (CRP), total cholesterol, low-density lipoprotein (LDL), albumin, uric acid, eGFR, creatinine, cholinesterase (ChE), total protein, mean corpuscular hemoglobin (MCH), Quick value, mean platelet volume (MPV), high-density lipoprotein (HDL), glycosylated hemoglobin (HbA1c). Subsequently, both the trauma surgery process data and the basic clinical data from the interview, which are shown in Table 2a and b, were compared with the score.

### Statistical analysis

Statistical analysis was performed using Prism (GraphPad Version 10, San Diego, CA, USA) and RStudio (Version 4.3.1, Boston, MA, USA). Results are presented as means ± standard error of mean, median, or percentages. A fixed alpha level of 0.05 was assumed as the threshold for statistical significance. A volcano plot was generated using multiple Mann–Whitney tests from the laboratory values, which were coded to 0 and 1. Due to multiple testing, an adjustment of the p-value was made to interpret the results. The patients were divided into six groups according to their score (0–0.1; 0.101–0.2; 0.201–0.3; 0.301–0.4; 0.401–0.5; > 0.5). Kaplan–Meier curves were created to examine the survival rates between the six groups. To assess the quality of the test, Receiver Operating Characteristic (ROC) curves and the corresponding AUC values were generated for each individual parameter, as well as for the FI-Lab44 and FI-Lab21 for the 6-month and 1-year endpoints. An AUC value of 0.9 is considered very good, 0.8 is considered good and 0.7 is considered useful. Cox proportional hazard models were used to determine the influence of score levels on survival. Hazard ratios (HRs) for the FI-Lab21 scores were analyzed separately and adjusted for age and gender to account for potential confounders. Spearman’s *ρ* was used for correlation analyses. Differences between the variables of the geriatric assessment and the trauma surgery process data were calculated using Mann–Whitney *U*-tests. The Mann–Whitney *U*-test, the Spearman correlation, and, where necessary, the chi-square test or Fisher’s exact test were used to determine possible correlations between the clinical parameters and the FI-Lab. The Shapiro–Wilk test was used to test for normal distribution.

**Table 1:** Laboratory parameters used and their reference values

Blood Parameter	Female	Male
Potassium (mmol/l)	3.7-5.1	3.7-5.1
Sodium (mmol/l)	135-145	135-145
Phosphate (mmol/l)	0.81-1.45	0.81-1.45
Calcium (mmol/l)	2.2-2.55	2.2-2.55
C-reactive protein (mg/l)	<6	<5
Creatinine (µmol/l)	45-84	59-104
eGFR* (mU/min)	>45	>45
Amylase (U/l)	<100	<100
Gamma-glutamyltransferase (U/l)	<38	55
Phosphatase, alkaline (U/I)	35-104	40-129
Cholesterol, total (mg/dl)	< 200	<200
HDL Cholesterol (mg/dl)	>45	>40
LDL Cholesterol (mg/dl)	<115	<115
non-HDL Cholesterol (mg/dl)	<150	<150
Triglycerides (mg/dl)	<190	<190
Protein, total (g/l)	65-80	65-80
Albumin (g/l)	35-52	35-52
Bilirubin (µmol/l)	2-21	2-21
Ferritin (ug/l)	27-365	27-365
Thyroid stimulating hormone (mU/l)	0.27-4.2	0.27-4.2
International normalized ratio	0.9-1.25	0.9-1.25
Quick value (%)	70-130	70-130
Activated partial thromboplastin time (sec)	26-36	26-36
Cholinesterase enzyme (kU/l)	4.26-11.25	5.32-12.92
Aspartate aminonotranferase (U/l)	<31	<35
Alanine transaminase (U/l)	<34	45
White blood cells (10³/µL)	3.6-10.5	3.6-10.5
Red blood cells (Mio./pl)	3.85-5.2	4-5.65
Hemoglobin (g/dl)	11.8-15.8	12.5-17.2
Hematocrit (%)	35-45.5	37-49
Red blood Cell Distribution Width (%)	11.5-15	11.5-15
Mean corpuscular volume (fl)	80-101	80-101
Mean corpuscular hemoglobin (pg)	27-34	27-34
Mean corpuscular hemoglobin concentration (g/dl)	31.5-36	31.5-36
Platelets (10³/µL)	160-370	160-370
Platelets >12fl (%)	15-35	15-35
Mean platelet volume (fl)	8.5-11.5	8.5-11.5
Platelet distribution width (fl)	9-17	9-17
HbA1c**(%)	4.8-5.6	4.8-5.6
HbA1c**(mmol/mol)	29-38	29-38
1.25 Vitamin D (pg/ml)	15.2-90.1	15.2-90.1
Bone alkaline phosphatase (g/l)	5-27	5.7-33
lactate dehydrogenase (U/l)	<248	<249
Uric acid (µmol/l)	140-340	200-420

*Estimated Glomerular Filtration Rate

**Glycated Hemoglobin

## Results

In the cohort, 249 patients underwent surgery for HF at HMS. Of these, 14 were excluded from the analysis, including 6 men and 8 women, with a mean age of 81.64 years (± 2.08), a BMI of 25.49 (± 1.23) and an ASA score of 2.57 (± 0.17). In 4 patients, less than 70% of the laboratory parameters were available, in 1 patient, no data could be accessed, in 3 patients, the second hip fracture was excluded, and in 6 patients, there was a loss to follow-up. Thus, 235 patients with a complete data set could be included in the analysis.

Of the 235 patients included, 67.34% were women. The mean age of the cohort was 83.65 years (± 0.41). A detailed characterization of the cohort can be found in Table 2a and b.

90 patients (38.3%) died within the first year after an average of 96 (± 11.03) days. Seventeen patients (18.88% of all decedents, 7.23% of the total cohort) died immediately after surgery during their inpatient stay.

**Table 2:** a: Baseline characteristics and surgical details of the cohort. b: Postoperative outcomes and functional status

Categories		All	Male	Female	*p* value	Alive	Dead	*p *value
Total number		235	77	158	**<0.0001**	145	90	-
Alive		145	43	102	-	-	-	-
Death within 12 months		90	34	56	-	-	-	-
Age (years)		83.65±0.41	82.35±0.64	84.28±0.51	**0.0206**	82.89±0.49	84.87±0.70	**0.0249**
BMI*		24.12±0.25	24.73±0.35	23.82±0.33	0.0568	24.09±0.32	24.18±0.42	0.7847
ASA**		2.67±0.04	2.70±0.07	2.66±0.05	0.5322	2.57±0.05	2.84±0.07	**0.0013**
Diagnosis	Femoral neck	116	37	79	0.8908	68	48	0.0929
	Trochanteric	108	37	71		74	34	
	Others	11	3	8		3	8	
Intervention	Hemiarthroplasty	102	35	67	06016	58	44	0.291
	Total-hip arthroplasty	10	2	8		9	1	
	Dynamics hip screw	8	1	7		6	2	
	Intramedullary nail	92	29	63		58	34	
	Others	22	9	13		14	8	
Time in emergency room (min)		295.86±10.80	285.19±18.28	300.91±13.37	0.4227	283.6±11.8	317.1±21.2	0.3619
Time to surgery (min)		1493.78±107.1	1600.99±225.5	1441.54±115.6	0.7756	1357±124.5	1713±193.4	0.0989
OP duration (min)		75.4±2.68	77.83±5.34	74.22±3.03	0.8587	72.95±3.35	79.34±4.5	0.289
Preoperative anticoagulation	None	90	23	67	0.3997	63	27	0.1292
	Antithrombotics	69	26	43		39	30	
	NOAC/NOAC+ASS	55	21	34		35	20	
	VKA***/VKA+ASS	6	2	4		3	3	
	Others	15	5	10		5	10	
Perioperative transfusions		0.25±0.05	0.39±0.11	0.18±0.05	0.1426	0.12±0.04	0.44±0.10	**0.0004**
Length of stay (days)		9.58±0.32	10.40±0.65	9.18±0.36	0.0477	9.24±0.28	10.12±0.71	0.371
Mortality (%)		38.3	44.2	35.4	0.2021	-	-	-
Time of survival (days)		262.1±9.52	241.89±17.32	269.5±11.36	0.3232	-	96.31±11.06	-
Place of discharge	Acute geriatics or rehabilitation	137	43	94	0.5949	94	43	**<0.0001**
	Nursing home	34	9	25		20	14	
	Death	17	7	10		0	17	
	Home	25	11	14		19	6	
	Others	22	7	15		12	10	
PMS**** at admission	Able to walk inside house (0-3)	2.32±0.06	2.32±0.11	2.32±0.07	0.8483	2.54±0.07	1.92±0.11	**<0.0001**
	Able to walk outside house (0-3)	1.92±0.08	2.07±0.13	1.84±0.10	0.165	2.14±0.10	1.53±0.13	**<0.0001**
	Able to go shopping (0-3)	1.59±0.10	1.75±0.17	1.51±0.12	0.2667	1.84±0.12	1.16±0.15	**0.0005**
	Sum (0-9)	5.83±0.22	6.19±0.39	5.63±0.27	0.0773	6.47±0.26	4.66±0.36	**<0.0001**
PMS after 1 year	Able to walk inside house (0-3)		2.0±0.1440	2.081±0.09489	0.5433	2.072±0.07819	-	-
	Able to walk outside house (0-3)	-	1.561±0.1713	1.670±0.1153	0.6446	1.650±0.08716	-	-
	Able to go shopping (0-3)	-	0.9756±0.1896	1.060±0.1153	0.6049	1.035±0.09832	-	-
	Sum (0-9)	-	4.650±0.4509	4.848±0.2826	0.5885	4.791±0.2387	-	-
Level of care at admission		1.41±2.15	1.42±0.22	1.41±0.13	0.7623	1.19±0.13	18.1±0.17	**0.002**
Level of care after 1 year		-	2.073±0.2736	2.208±0.1575	0.6766	2.170±0.1376	-	-

*Body-Mass-Index

**American Society of Anesthesiologists

***Vitamin-Kantagonist

****Parker Mobility Score

**Fig. 1:**
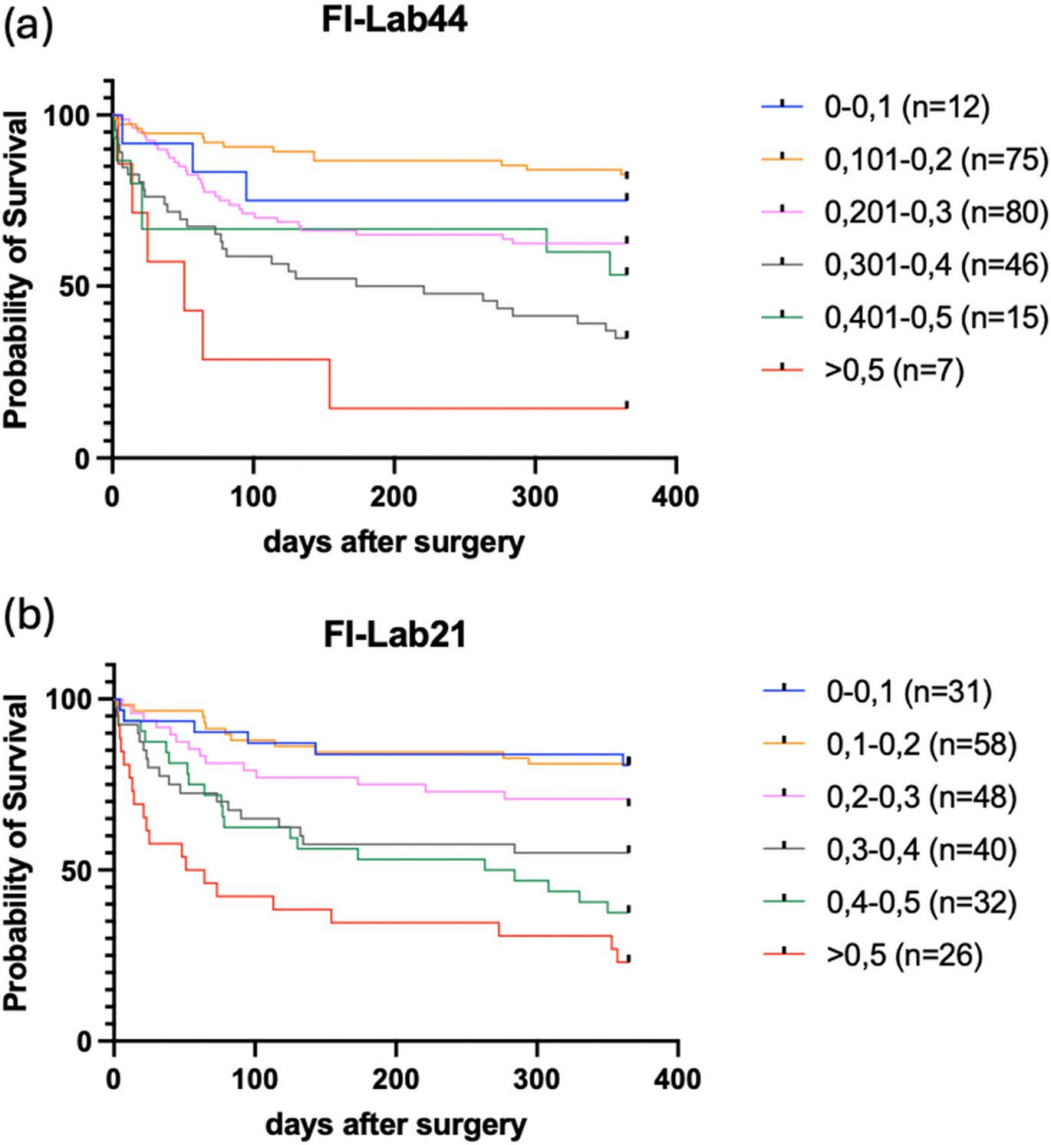
**a**, **b**: Kaplan Meier survival curves at 12 months for the FI-Lab44 (**a**) and the FI-Lab21 (**b**)

**Fig. 2:**
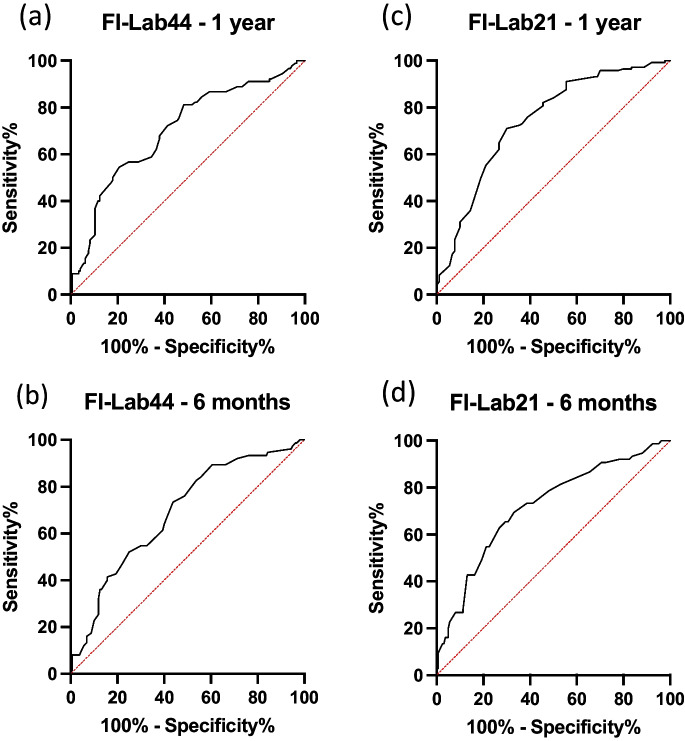
**a**, **b**, **c**, **d**: ROC curves of the FI-Lab44 for 1 year (AUC 0.7025) and for 6 months (AUC 0.6870), the FI-Lab21 for 1 year (AUC 0.7423) and for 6 months (AUC 0.7177)

The FI-Lab44 has already demonstrated significant Kaplan–Meier survival curves for the 6 groups (*p* < 0.0001), as well as an acceptable ROC curve with an AUC of 0.7025. The correlation of the score with survival time also showed significant results (*p* < 0.0001). This analysis showed that there is a correlation between the laboratory parameters and the 1-year outcome (see Figs. 1a and 2a, b).

**Fig. 3:**
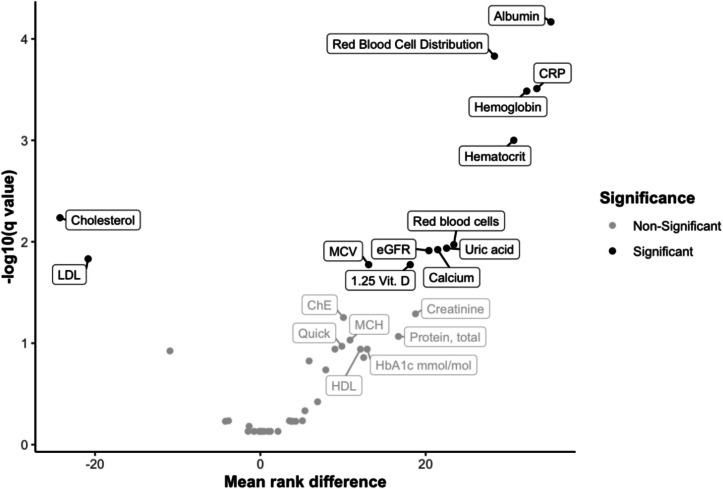
Volcano plot of the 44 parameters with labeling of the 21 selected parameters of the FI-Lab21

As a score of 44 parameters may not be practicable in everyday clinical practice, the next step was to create a compressed score from the 21 most relevant parameters. The volcano plot showed a significant difference in the number of deviations between deceased and surviving patients for 6 parameters, which were thus identified as particularly relevant. These were: albumin, erythrocyte size variability, CRP, hemoglobin, hematocrit, and total cholesterol (See Fig. 3).

Despite the low significance of the individual parameters in the ROC analysis and in the Volcano plot, the total score for the 1-year and 6-month outcome proved to be meaningful (see Table 3).

**Table 3:** Parameters of the FI-Lab21 and the corresponding AUC values for the period of 1 year and 6 months

	1 year				6 months			
Lab Parameter	Area	Std Error	95%Kl	p Wert	Area	Std Error	95%Kl	p Wert
Albumin	0,6515	0,03813	0,5768 to 0,7263	**0,0001**	0,6297	0,04037	0,5506 to 0,7089	**0,0015**
C-reactive protein	0,6423	0,03702	0,5698 to 0,7149	**0,0002**	0,5915	0,03961	0,5138 to 0,6691	**0,0239**
Hemoglobin	0,6372	0,03764	0,5634 to 0,7109	**0,0004**	0,621	0,03962	0,5434 to 0,6987	**0,0028**
Hematocrit	0,6305	0,03746	0,5570 to 0,7039	**0,0008**	0,6133	0,03934	0,5362 to 0,6904	**0,0051**
Red Blood Cell Distribution Width	0,6205	0,0389	0,5443 to 0,6967	**0,0019**	0,601	0,04123	0,5202 to 0,6818	**0,0126**
Total Cholesterol	0,6045	0,03731	0,5314 to 0,6777	**0,0074**	0,572	0,03923	0,4951 to 0,6489	0,0763
Uric acid	0,5997	0,03948	0,5223 to 0,6771	**0,0123**	0,5743	0,0415	0,4930 to 0,6557	0,073
Red Blood Cells	0,5996	0,03845	0,5243 to 0,6750	**0,0103**	0,6033	0,0402	0,5245 to 0,6821	0,0107
Calcium	0,5945	0,03947	0,5172 to 0,6719	**0,0169**	0,5926	0,04124	0,5117 to 0,6734	0,0244
LDL cholesterol	0,5898	0,03769	0,5159 to 0,6637	**0,0215**	0,5555	0,03966	0,4777 to 0,6332	0,172
eGFR	0,5868	0,03893	0,5105 to 0,6631	**0,0254**	0,5542	0,04094	0,4739 to 0,6344	0,1809
Cholinesterase enzyme	0,5844	0,05644	0,4738 to 0,6950	0,129	0,5618	0,06019	0,4438 to 0,6797	0,2937
1.25 Vitamin D	0,5828	0,0405	0,5034 to 0,6622	0,04	0,5813	0,04247	0,4980 to 0,6645	0,0526
Creatine	0,5799	0,03836	0,5047 to 0,6551	0,0396	0,5577	0,04025	0,4788 to 0,6366	0,154
Total Protein	0,572	0,03865	0,4962 to 0,6477	0,066	0,5798	0,04003	0,5013 to 0,6583	0,0502
HbA1c mmol/mol	0,563	0,04121	0,4822 to 0,6438	0,1267	0,5783	0,04295	0,4941 to 0,6625	0,0681
Mean corpuscular volume (MCV)	0,5557	0,03936	0,4786 to 0,6329	0,151	0,5358	0,04119	0,4551 to 0,6166	0,3761
HDL Cholesterol	0,5524	0,03941	0,4752 to 0,6297	0,1802	0,5668	0,04127	0,4859 to 0,6477	0,1003
Mean corpuscular hemoglobin (MCH)	0,5462	0,03925	0,4692 to 0,6231	0,2343	0,5402	0,04118	0,4595 to 0,6209	0,3206
Quick value	0,542	0,03925	0,4650 to 0,6189	0,2798	0,5527	0,04137	0,4716 to 0,6338	0,1929
Mean platelet volume	0,5386	0,0394	0,4613 to 0,6158	0,3224	0,5467	0,04155	0,4653 to 0,6281	0,2508

The FI-Lab21 was not normally distributed with a mean of 0.3 (± 0.011), a minimum of 0 and a maximum of 0.85. The median was 0.28. The 1st, 5th, 95th and 99th percentiles were 0, 0.05, 0.6 and 0.8, respectively.

For 1-year survival, the FI-Lab21 achieved an acceptable accuracy with an AUC of 0.7423. The Kaplan–Meier survival curves for the 6 groups already categorized were also significant (*p* < 0.0001) (see Figs. 1b and 2c).

For 6-month survival, the score achieved an AUC of 0.7177. The individual laboratory parameters also performed worse in the ROC analysis than for 1 year (see Fig. 2d). Nevertheless, the score was significant in the Kaplan–Meier analysis and achieved an acceptable AUC. It is therefore also a relevant predictor for the outcome after 6 months.

The evaluation of the Cox proportional hazard ratio revealed a significant increase in the risk of death both for the period of 1 year and 6 months from a score of 0.4 and higher. Compared to a score of 0–0.1, the risk increases 6.5 times higher from a score of 0.501 for the period of 12 months (see Table 4).

**Table 4:** a, b: Hazard ratios of FI-Lab21 for survival after 12 months (a) and 6 months (b).

Hazard ratios	Variable	Estimate	95% Cl (profile likelihood)	*p* value
**12 months**				
exp(β1)	group [2]	0.9472	0.3601 to 2.751	0.915
exp(β2)	group [3]	1.717	0.6978 to 4.816	0.2631
exp(β3)	group [4]	2.799	1.171 to 7.729	0.0293
exp(β4)	group [5]	3.928	1.667 to 10.77	0.0034
exp(β5)	group [6]	6.507	2.751 to 17.89	<0.0001
exp(β6)	age	1.034	1.000 to 1.070	0.0531
exp(β7)	gender [2]	1.267	0.8136 to 1.946	0.2856
**6 months**				
exp(β1)	group [2]	0.943	0.3255 to 3.071	0.9162
exp(β2)	group [3]	1.610	0.5972 to 5.062	**0.3708**
exp(β3)	group [4]	3.039	1.200 to 9.258	**0.0292**
exp(β4)	group [5]	3.039	1.170 to 9.387	**0.0323**
exp(β5)	group [6]	5.515	2.154 to 16.92	**0.0009**
exp(β6)	age	1.043	1.005 to 1.083	**0.0285**
exp(β7)	gender [2]	1.823	1.134 to 2.910	**0.0122**

### Trauma surgery process data

Mortality was not associated with gender, BMI, fracture type, type of surgery, duration of surgery, or use and type of preoperative anticoagulation. Furthermore, there were no differences between the deceased and the survivors regarding the time to surgery, LOS, or the time in the emergency room.

Deceased patients were older and had higher scores for preoperative level of care, number of transfusions administered perioperatively, and ASA score.

Higher FI-Lab21 Scores were associated with significantly increased 1-year mortality and male gender (mean men: 0.34 ± 0.02, mean women: 0.28 ± 0.01, *p* = 0,0446).

The ASA score of the patients (*p* = 0.0003), the time to surgery (*p* = 0.0131), the number of perioperative performed transfusions (*p* < 0.0001) and the postoperative complication rate (*p* = 0.0252) also increased with increasing score.

Patients with a higher preoperative score also had lower values for mobility in the PMS at home on admission (*p* = 0.0019). No differences were found for mobility outside the home and shopping. No correlation was found between the score and the patient's age, BMI, height, duration of surgery, LOS, or level of care.

### Interviews

Patients with a higher baseline FI-Lab21 score achieved significantly lower scores in quality of life (EQ-5D-5L) (*p* = 0.0389) and mobility inside (*p* = 0.0399) and outside the home (*p* = 0.0163) 1 year postoperatively assessed with the PMS.

Within 1 year, there was a decrease in the sum of the PMS among the survivors from a preoperative mean of 5.83 to a mean of 4.79 after 1 year (*p* < 0.0001). The level of care among survivors increased from a mean of 1.12 ± 0.1303 preoperatively to a mean of 2.17 ± 0.1376 postoperatively (*p* < 0.0001).

The level of care and the number of patients who required help with washing and dressing, mobility, anxiety/depression, shopping, self-care, as well as the frequency and intensity of pain, showed no association with the score.

## Discussion

### Brief introduction and main findings

This retrospective cohort study aimed to develop a laboratory-based frailty index (FI-Lab21) for risk prediction of older patients with HF. The FI-Lab21 was developed using routinely collected laboratory parameters and aimed to identify patients with an increased risk of mortality within 12 months after inpatient treatment in the department of trauma surgery. The study found a clear correlation between a higher score and increased mortality within the first postoperative year, providing a significant prediction for the 1-year survival of patients with HF. The FI-Lab21 achieved an AUC of 0.7423 for 1-year survival and significant differences in the Kaplan–Meier survival curves for the six categorized subgroups.

The study is characterized by the homogeneity of the patient group by clinical diagnosis and low exclusion rate, especially the low loss to follow-up rate. By using purely routine parameters, it is cost-effective, time-efficient, and can be carried out by lay personnel. Patients do not have to actively contribute to its assessment.

### Significance

The results of the FI-Lab21 correlate with other established geriatric and trauma surgery risk instruments. An increased ASA score, reduced PMS, and longer time to surgery are associated with an increased FI-Lab21, indicating an increased vulnerability and poorer general health of the patients. The study reveals that patients with frailty syndrome often experience increased perioperative bleeding tendency or pre-existing anemia, which is often associated with frailty syndrome and significantly impacts their postoperative course [33, 34]. Hemoglobin, albumin, total protein and vitamin D, which stood out in this study, are crucial in diagnosing malnutrition, which is often associated with frailty syndrome and affects postoperative outcomes [35–38]. Albumin was found to be the most relevant parameter in the study. Mobility and independence deteriorate significantly after a femoral neck fracture, as observed in this study [39]. The Parker Mobility Score decreased by 1.04 points within 1 year, while care level increased by 1.05 points over the same period.

### Comparison to other models

The current gold standard for diagnosing frailty syndrome is screening scores that incorporate geriatric assessments, such as the phenotype approach by Fried et al. or the accumulation of deficits approach by Mitnitski et al. [19, 20]. However, these scores are not applicable in clinical settings due to the increasing incidence of functional limitations and cognitive decline with age. The Nottingham Hip Fracture Score, developed specifically for hip fractures, includes hemoglobin as the only laboratory parameter, achieved AUC values of 0.71 for 30-day survival [21].

Previous studies have compared frailty scores composed of geriatric assessments with purely laboratory-based scores, showing that laboratory-based frailty scores provide equivalent results and can be used as valid instruments for risk prediction in older patients [22–25, 40]. Jäger et al. also developed an FI-Lab based on 20 blood and one urine parameter, which achieved AUC values of 0.72 for both 6-month and 1-year survival [22].

The FI-Lab21 study is unique in its statistically calculated selection of laboratory parameters and its use of data from a specific group of patients who have all received the same diagnosis and treatment. This approach allows for more accurate risk prediction, especially in frailty syndrome, which requires consideration of the specific patient group and situational circumstances.

### Limitations

The present study has limitations, including its single center setting, the specific laboratory profile used to create the score, the retrospective nature of the study, and the absence of a gold standard to verify the results as well as we focused on patients with HF. Some parameters, such as 1.25 Vitamin D, ChE, and bone alkaline phosphatase, were only available in a few patients, which could limit the statistical significance of these values. As many patients in this study had cognitive limitations, they were unable to answer the questions in the interview themselves, which could lead to a possible bias. Patients’ previous illnesses, comorbidities and medication were not recorded in this study, which could have helped to substantiate the validity of the FI-Lab21. Additionally, many of the variables used in the frailty index (FI-Lab) may be influenced by the acute inflammatory response triggered by the fracture event, rather than reflecting the degree of frailty of the subject itself. Some laboratory values are related to nutritional status, which is often influenced by frailty and dementia, making it difficult to fully differentiate these factors.

### Future research

Future research should focus on further deepening these aspects and increasing the validity of the FI-Lab21. Upcoming projects should consider AI-based analyses with a larger cohort to calculate the exact influence of individual parameters on outcomes. Using fewer parameters could create a more precise formula that categorizes parameters according to their actual influence, enabling more accurate predictions about survival.

Targeted strategies should be developed to offer patients with high frailty scores individualized multidisciplinary treatments. These include, for example, the targeted treatment of malnutrition and the implementation of an adapted rehabilitation program. This could improve patients’ quality of life and save the healthcare system monetary and human resources [41].

## Conclusion

The present study represents a promising approach to the development of a laboratory-based frailty index for the risk prediction of older patients with hip fractures. The results illustrate the clinical relevance and validity of the developed score as a predictor of postoperative outcome and emphasize the importance of differentiated risk stratification in perioperative care, so that the FI-Lab21 could serve as a practical tool for identifying high-risk patients in trauma surgery. Compared to other studies, it is characterized in particular by a simple and in many respects resource-saving calculation.

## Data Availability

Anonymized data are available upon request.
